# Both PKA and Epac Pathways Mediate N-Acetylcysteine-Induced Connexin43 Preservation in Rats with Myocardial Infarction

**DOI:** 10.1371/journal.pone.0071878

**Published:** 2013-08-28

**Authors:** Tsung-Ming Lee, Shinn-Zong Lin, Nen-Chung Chang

**Affiliations:** 1 Department of Medicine, Cardiology Section, Tainan Municipal An-Nan Hospital-China Medical University, Tainan, Taiwan; 2 Department of Medicine, China Medical University, Taichung, Taiwan; 3 Department of Internal Medicine, School of Medicine, College of Medicine, Taipei Medical University, Taipei, Taiwan; 4 Neuropsychiatry Center, China Medical University Hospital, Taichung, Taiwan; 5 Department of Neurosurgery, Taina Municipal An-Nan Hospital-China Medical University, Tainan, Taiwan; 6 Division of Cardiology, Department of Internal Medicine, Taipei Medical University Hospital, Taipei, Taiwan; 7 Graduate Institute of Immunology, China Medical University, Taichung, Taiwan; 8 Department of Neurosurgery, China Medical University Beigan Hospital, Yunlin, Taiwan; University Heart Center Freiburg, Germany

## Abstract

Cardiac remodeling was shown to be associated with reduced gap junction expression after myocardial infarction. A reduction in gap junctional proteins between myocytes may trigger ventricular arrhythmia. Therefore, we investigated whether N-acetylcysteine exerted antiarrhythmic effect by preserving *connexin43* expression in postinfarcted rats, focusing on cAMP downstream molecules such as protein kinase A (PKA) and exchange protein directly activated by cAMP (Epac). Male Wistar rats after ligating coronary artery were randomized to either vehicle, or N-acetylcysteine for 4 weeks starting 24 hours after operation. Infarct size was similar between two groups. Compared with vehicle, cAMP levels were increased by N-acetylcysteine treatment after infarction. Myocardial *connexin43* expression was significantly decreased in vehicle-treated infarcted rats compared with sham operated rats. Attenuated *connexin43* expression and function were blunted after administering N-acetylcysteine, assessed by immunofluorescent analysis, dye coupling, Western blotting, and real-time quantitative RT-PCR of connexin43. Arrhythmic scores during programmed stimulation in the N-acetylcysteine-treated rats were significantly lower than those treated with vehicle. In an *ex vivo* study, enhanced connexin43 levels afforded by N-acetylcysteine were partially blocked by either H-89 (a PKA inhibitor) or brefeldin A (an Epac-signaling inhibitor) and completely blocked when H-89 and brefeldin A were given in combination. Addition of either the PKA specific activator N6Bz or Epac specific activator 8-CPT did not have additional increased connexin43 levels compared with rats treated with lithium chloride alone. These findings suggest that N-acetylcysteine protects ventricular arrhythmias by attenuating reduced *connexin43* expression and function via both PKA- and Epac-dependent pathways, which converge through the inactivation of glycogen synthase kinase-3β.

## Introduction

Cardiac remodeling was shown to be associated with gap junction heterogeneities after myocardial infarction (MI) [1]. A dysfunction of the cardiac gap junction, which contributes to electrical cell-to-cell coupling, is one of essential factors known to generate arrhythmias. These channels permit molecules with molecular masses of less than 1 kDa, such as small metabolites, ions, and intracellular signaling molecules (i.e., glutathione [GSH], cyclic 3′,5′-adenosine monophosphate [cAMP]), to pass through [2]. Connexin43 (Cx43) is the 43-kDa member of a conserved family of membrane spanning gap junction proteins, of which Cx43 is the principal junctional protein in mammalian myocardium [3]. A reduction in gap junctional coupling between myocytes may be an important morphological feature that could interact with altered membrane properties in diseased myocardium [4]. Decreased ventricular Cx43 levels have been implicated in the pathogenesis of ventricular arrhythmias in humans [5] and knockout mice [6]. In particular the 1b phase of ischemia-induced arrhythmias, which often terminates in ventricular fibrillation and thus is responsible for sudden cardiac death, is thought to result from the uncoupling of gap junction [7]. Recently, Cx43 gene transfer has been shown to attenuate arrhythmia susceptibility in the healed border zone after MI [8].

cAMP is a modulator of junctional permeability in heart muscle [9]. cAMP increases Cx43 mRNA [10]. The main intracellular receptor of cAMP is cAMP-dependent protein kinase (PKA), which can phosphorylate a number of substrates upon activation. 8-bromoadenosine 3′:5′-cyclic monophosphate (8-Br-cAMP), a PKA activator, enhanced transjunctional conductance (*g*j) and PKA inhibitors suppressed *g*j in neonatal and adult rat ventricular cardiomyocytes [11], [12]. The intercellular communication through Cx43 is accelerated by its phosphorylation on Ser364 by PKA [13]. Cx43 with a ser364pro mutation displayed an altered response to PKA-related signaling cascades [14]. While most studies of cAMP signaling have focused on PKA, cAMP has been shown to regulate gene transcription, cellular proliferation, and cytokine signaling through PKA-independent pathway [15]. Previous studies have shown that exchange protein directly activated by cAMP (Epac) is associated with increased expression of Cx43 in neonatal rat cardiomyocytes [9]. Epac belongs to a family of cAMP regulated guanine nucleotide exchange factors that mediate PKA-independent signal transduction properties of the second messenger cAMP [16]. Epac has been shown to either antagonize [17] or synergize with PKA [18]. *N*-acetylcysteine (NAC) is a thiol-containing compound that serves as a GSH precursor [19]. Many effects of GSH are caused by induction of a second messenger molecule, cAMP [20], probably via activation of adenylate cyclase and subsequent increase in intracellular cAMP. Although most of the biologic actions of cAMP are supposed to be mediated by PKA signals, it remains unknown whether PKA and Epac collaborate in modulating cAMP-induced Cx43 expression upon NAC action. Furthermore, the engagement of distinct cAMP-regulated effectors has not been yet addressed in the infarcted heart. Thus, we assessed (1) whether NAC administration after infarction modulates Cx43 expression and attenuates ventricular arrhythmias, and (2) whether NAC-induced Cx43 changes are PKA or Epac dependent in a rat MI model by the use of inhibitors of PKA, Epac and adenylate cyclase as well as the measurement of cyclic nucleotides. In this report, we show that Cx43 activated through the inactivation of glycogen synthase kinase-3β (GSK-3β) by NAC-induced cAMP-PKA and cAMP-Epac signaling attenuated ventricular arrhythmias after MI.

## Methods

### Animals

The animal experiment was approved and conducted in accordance with local institutional guidelines of the China Medical University for the care and use of laboratory animals (Permit Number: 102-62-N) and conformed with the *Guide for the Care and Use of Laboratory Animals* published by the US National Institutes of Health (NIH Publication No. 85-23, revised 1996).

#### Experiment 1 (*in vivo study*)

Male Wistar rats aged 8 weeks (300–350 g) were subjected to ligation of the anterior descending artery as previously described [21] resulting in infarction of the LV free wall. In brief, to create the model, rats were anesthetized with ketamine-xylazine (90 mg/kg-9 mg/kg, intraperitoneally). After adequate anesthesia they were intubated with a 14-gauge polyethylene catheter and ventilated with room air using a small animal ventilator (model 683, Harvard Apparatus, Boston, MA). The heart was exposed via a left-sided thoracotomy, and the anterior descending artery was ligated using a 5-0 silk between the pulmonary outflow tract and the left atrium. Sham rats underwent the same procedure except the suture was passed under the coronary artery and then removed. Rats were randomly assigned into either vehicle (saline) group or NAC (500 mg/kg per day, Sigma, St Louis, MO, USA). The dose of NAC used in this study has been shown to effectively modulate cardiac GSH without significantly changing blood pressure [22].

The drug was started 24 hours after infarction, at a time when it could produce maximum benefits [23]. The study duration was designed to be 4 weeks because the majority of the myocardial remodeling process in the rat (70–80%) is complete within 3 weeks [24]. The drugs were administered by daily oral gavage. The drug was withdrawn about 24 hours before the end of the experiments in order to eliminate its pharmacological actions.

#### Experiment 2 (*ex vivo study*)

To test the relative importance of PKA and Epac in NAC-related Cx43 levels, we used inhibitors of adenylate cyclase, PKA, and Epac in an *ex vivo* model. Four weeks after induction of MI by coronary ligation, infarcted rat hearts were excised and Langendorff-perfusion with a noncirculating modified Tyrode's solution was previously described [21]. The hearts were subjected to no treatment (vehicle), NAC (60 mM), NAC+SQ-22536 (80 µM, an adenylate cyclase inhibitor), NAC+H-89 (0.1 µM, a highly specific inhibitor of PKA), NAC+brefeldin A (100 µM, an Epac-signaling inhibitor), and NAC+H-89+brefeldin A. A confirmation of the participation of Epacs was obtained with the use of brefeldin A [25]. The doses of SQ-22536, H-89, and brefeldin A have been shown to be effective in modulating biological activities [26]. SQ-22536, H-89, and brefeldin A were all from Sigma (St Louis, MO, USA). Drugs were perfused for 60 minutes. At the end of the study, all hearts (n = 10 in each group) were used for performing Cx43 protein and dye coupling measurement at the border zone (<2 mm within the infarct).

#### Experiment 3 (*ex vivo study*)

To further dissect the downstream pathways of PKA and Epac, we assessed the role of GSK-3β on Cx43 levels and GSK-3β inhibitor lithium chloride (LiCl) was used to suppress GSK-3β activity. Infarcted rat hearts four weeks after induction of MI by coronary ligation were isolated and Langendorff-perfusion was performed as Experiment 2. The hearts were subjected to no treatment (vehicle), N6-benzoyladenosine 3′,5-cyclic monophosphate (1 mM, N6Bz, a PKA specific activator, BIOLOG Life Science Institute), 8-(4-chlrorophenylthio)-2′-O-methyladenosine-3′,5′-cyclic monophosphate) (1 mM, 8-CPT, an Epac specific activator, Calbiochem), LiCl (20 mM, a GSK-3β inhibitor; Sigma–Aldrich, Inc., St. Louis, MO, USA), LiCl+N6Bz, and LiCl+8-CPT. The doses of N6Bz, 8-CPT, and LiCl have been shown to be effective in modulating biological activities [9], [27]. Each heart was perfused with the same protocol as Experiment 2. Western blot and dye coupling measurement from the border zone were performed.

### Hemodynamics and Infarct size measurements

Hemodynamic parameters were measured in anesthetized rats with an intraperitoneal dose of ketamine-xylazine (90 mg/kg-9 mg/kg) at the 28^th^ day after operation. A polyethylene Millar catheter was inserted into the LV and connected to a transducer (Model SPR-407, Miller Instruments, Houston, TX) to measure LV systolic and diastolic pressure as the mean of measurements of five consecutive pressure cycles as previously described [21]. The maximal rate of LV pressure rise (+dP/d*t*) and decrease (−dP/d*t*) were measured. After the arterial pressure measurement, the electrophysiological tests were performed. At completion of the electrophysiological tests, the atria and the right ventricle were trimmed off, and the LV was rinsed in cold physiological saline, weighed, and immediately frozen in liquid nitrogen after obtaining a coronal section of the LV for infarct size estimation. A section, taken from the equator of the LV, was fixed in 10% formalin and embedded in paraffin for determination of infarct size. Each section was stained with hematoxylin and eosin, and trichrome. The infarct size was determined as previously described [28]. With respect to clinical importance, only rats with large infarction (>30%) were selected for analysis.

### *In Vivo* Electrophysiological Studies

To assess the potential arrhythmogenic risk of Cx43, we performed *in vivo* programmed electrical stimulation after left thoracotomy and artificial respiration. Because the residual neural integrity at the infarct site is one of the determinants of the response to electrical induction of ventricular arrhythmias [29], only rats with transmural scar were included. Body temperature was maintained at 37°C with a thermostatically controlled heating lamp. Programmed electrical stimulation was performed with electrodes sewn to the epicardial surface of the right ventricular outflow tract. Pacing pulses were generated from a Bloom stimulator (Fischer Imaging Corporation, Denver, CO, USA). To induce ventricular arrhythmias, pacing was performed at a cycle length of 150 ms (S_1_) for eight beats, followed by one to three extrastimuli (S_2_, S_3_, and S_4_) at shorter coupling intervals. The endpoint of ventricular pacing was induction of ventricular tachyarrhythmia. Ventricular tachyarrhythmias including ventricular tachycardia and ventricular fibrillation were considered nonsustained when it lasted ≤15 beats and sustained when it lasted >15 beats. An arrhythmia scoring system was modified as previously described [24]. 0, noninducible preparations; 1, nonsustained tachyarrhythmias induced with three extrastimuli; 2, sustained tachyarrhythmias induced with three extrastimuli; 3, nonsustained tachyarrhythmias induced with two extrastimuli; 4, sustained tachyarrhythmias induced with two extrastimuli; 5, nonsustained tachyarrhythmias induced with one extrastimulus; 6, sustained tachyarrhythmias induced with one extrastimulus; and 7, tachyarrhythmias induced during the eight paced beats. If the heart stopped before the pacing, the arrhythmia score assigned to that heart was 8. When multiple forms of arrhythmias occurred in one heart, the highest score was used. The experimental protocols were typically completed within 10 minutes.

### Determination of gap junction permeability

Gap junction permeability at the border zone was determined by using Lucifer yellow according to the method of Ruiz-Meana et al [30]. with slight modification. In brief, the LV at the border zone was excised and an incision was quickly made in the endocardium. The LV was then immediately soaked and incubated in Krebs-Henseleit buffer containing Lucifer yellow (2.5 mg/ml) and rhodamine-conjugated dextran (2.5 mg/ml). The Lucifer yellow- and rhodamine-labeled regions were quantitatively measured from 10 randomly selected fields at a magnification of 100× by computerized planimetry (Image Pro Plus, Media Cybernetics, Silver Spring, M D., USA). The value was expressed as the ratio of the difference between Lucifer yellow-labeled area and rhodamine-labeled area to rhodamine-labeled area. The slides were coded so that the investigator was blinded to the rat identification.

### Real-time RT-PCR of Cx43

Real-time quantitative reverse transcription-polymerase chain reaction (RT-PCR) was performed from samples obtained from the border zone with the TaqMan system (Prism 7700 Sequence Detection System, PE Biosystems) as previously described [21]. For Cx43, the primers were 5′-TGAAAGAGAGGTGCCCAGACA-3′ (sense) and 5′-CGTGAGAGATGGGGAAGGACT-3′ (antisense). For cyclophilin, the primers were 5′-ATGGTCAACCCCACCGTGTTCTTCG-3′ and 5′-CGTGTGAAGTCACCACCCTGACACA-3′. Cyclophilin mRNA was chosen as the internal standard because it is expressed at a relatively constant level in virtually all tissues. Standard curves were plotted with the threshold cycles versus log template quantities. For quantification, Cx43 level was normalized to the expressed cyclophilin. Reaction conditions were programmed on a computer linked to the detector for 40 cycles of the amplification step. The results of the real-time RT-PCR assay were expressed as the Ct value, defined as the cycle number at which the sample passed a fixed fluorescence threshold and became positive. For each cDNA sample, the Ct value of the reference gene cyclophilin was subtracted from the Ct value of the sample to obtain ΔCt. Relative expression levels were calculated using the formula ΔΔCt = ΔCt (treated)−ΔCt (sham), and the value used to plot the relative level was calculated using the expression 2^−ΔΔCt^. Experiments were replicated three times and results expressed as the mean value.

### Western Blot Analysis of Cx43and GSK-3β

Samples obtained from the border zone (from either *in vivo* or *ex vivo* studies) were homogenized and incubated with rabbit polyclonal anti-Cx43 (Zymed, 71-0700), anti-GSK-3β, and anti-p-GSK-3β (Cell Signaling Technology, Beverly, MA) antibodies at 1∶500 dilution. The rabbit anti-Cx43 antibody was raised against a peptide corresponding to a segment of the third cytoplasmic domain (C-terminal portion) of rat Cx43 and can detect both phosphorylated (P1) and non-phosphorylated (P0) Cx43. The P1/P0 ratio was used as the magnitude of the phosphorylation of Cx43. Antigen-antibody complexes were detected with 5-bromo-4-chloro-3-indolyl-phosphate and nitroblue tetrazolium chloride (Sigma products, St. Louis, MO).

### Immunofluorescent Studies of Cx43

In order to investigate the spatial distribution and quantification of Cx43, analysis of immunofluorescent staining was performed on LV muscle from the border zone. Paraffin-embedded sections were performed at a thickness of 5 µM. Tissues were incubated with Chemicon polyclonal anti-Cx43 antibodies. The second antibody was monoclonal goat anti-mouse IgG conjugated to fluorescein isothiocyanate (Sigma), at 1∶50 dilution in PBS containing 0.5% BSA for 1 hour. Isotype-identical directly conjugated antibodies served as a negative control.

The density of Cx43-labeled areas was qualitatively estimated from 10 randomly selected fields at a magnification of 400×. The Cx43 density was measured on the tracings by computerized planimetry (Image Pro Plus, CA) as described previously [31]. The value was expressed as the ratio of Cx43-labeled area to total area. The slides were coded so that the investigator was blinded to the rat identification.

### ELISA for cAMP measurements

Myocardial cAMP was measured using enzyme-linked immunoassay kit (R&D Systems, Abingdon, UK) in myocardial homogenates from the border zone according to the manufacturer's instructions. The protein content was determined by the BCA protein assay kit and cAMP levels were expressed as µmol/g protein.

### Statistical Analysis

Results were presented as mean ± SEM. Statistical analysis was performed using the SPSS statistical package (SPSS, version 11.0, Chicago, Illinois). Differences among the groups of rats were tested by a one-way ANOVA. Subsequent analysis for significant differences between the two groups was performed with a multiple comparison test (Scheffe's method). Electrophysiological data (scoring of programmed electrical stimulation-induced arrhythmias) were compared by a Kruskal-Wallis test followed by a Mann-Whitney test. The significant level was assumed at value of p<0.05.

## Results

### NAC improved ventricular remodeling and cAMP levels

Differences in mortality between the infarcted groups were not found throughout the study in an *in vivo* study (data not shown). NAC had little effect on cardiac gross morphology in the sham-operated rats (data not shown). LV end-systolic pressure, LV end-diastolic pressure, and infarct size did not differ between the two infarcted groups (Table 1). Four weeks after infarction, the infarcted area of the LV was very thin and was totally replaced by fully differentiated scar tissue. The vehicle-treated infarcted group had an increase in right ventricular weight/body weight ratio and lung weight/body weight ratio, compared with NAC-treated infarcted group. The weight of the LV inclusive of the septum remained essentially constant for 4 weeks among the infarcted groups.

**Table 1:** Cardiac morphology, hemodynamics, and cAMP levels at the end of study.

		Infarction treated with	
Parameters	Sham	Vehicle	NAC
No. of rats	12	10	12
Body weight, g	380±4	375±5	395±4
HR, bpm	398±5	405±4	408±6
LVESP, mm Hg	114±2	104±3	115±3
LVEDP, mm Hg	5±1	18±1*	15±1*
+dp/d*t*, mm Hg/s	7462±102	2981±100*	4412±76* †
−dp/d*t*, mm Hg/s	6522±83	2501±83*	3082±83* †
Infarct size, %	…	40±1	40±1
LVW/BW, mg/g	2.20±0.05	3.05±0.11*	3.12±0.11*
RVW/BW, mg/g	0.48±0.03	0.72±0.04*	0.62±0.03* †
LungW/BW, mg/g	4.03±0.11	5.56±0.11*	4.58±0.11* †
cAMP, µmol/g protein	3.95±0.30	1.65±0.30*	2.87±0.32* †

Values are mean ± SEM. BW, body weight; HR, heart rate; LungW, lung weight; LVEDP, left ventricular end-diastolic pressure; LVESP, left ventricular end-systolic pressure; LVW, left ventricular weight; RVW, right ventricular weight.

* p<0.05 compared with sham;

† p<0.05 compared with the vehicle-treated group.

Compared with sham, ventricular remodeling after MI was associated with a significant reduction in cAMP content (3.95±0.30 in sham vs. 1.65±0.30 µmol/g protein, p<0.001, Table 1). Treatment with NAC had a significant increase of cAMP content compared with vehicle.

### NAC preserved Cx43 immunofluorescent density

**Figure 1:**
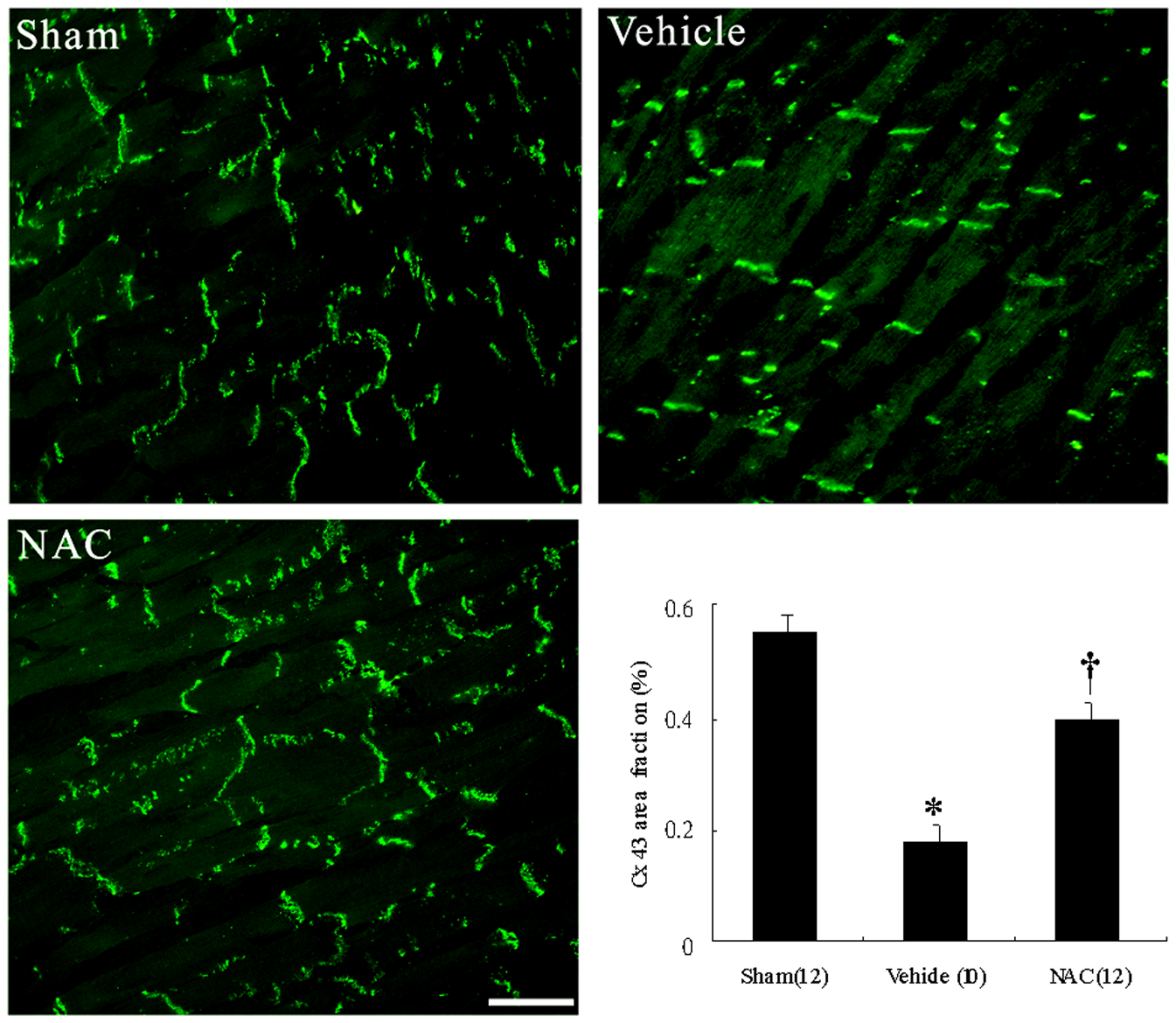
Representative immunofluorescent staining for Cx43. In sham, Cx43 forms clusters of punctate immunofluorescence domains confined to well-organized intercalated discs running across the longitudinal axis (magnification 400×). In contrast, infarction markedly decreases Cx43 proteins from the border zone. After administering NAC, Cx43 signals are increased. Bar = 50 µm. The proportion of the total cell area occupied by Cx43 immunoreactive signal at the border zone. Each column and bar represents mean ± SEM. *p<0.05 compared with sham and NAC-treated group; †p<0.05 compared with sham.

In the sham group, sections stained with the Cx43 antibody produce intense punctate labelling primarily at intercalated discs between cardiomyocytes (Figure 1), consistent with the gap junctions. Infarction results in a significant decrease in total amount of Cx43 immunoreactive signals of 67%. These signals at intercalated discs were significantly higher in animals treated with NAC. The ratio of Cx43 area to the total area was significantly increased by 40% in NAC-treated rats than that in vehicle-treated rats.

### NAC increased Cx43 levels

**Figure 2:**
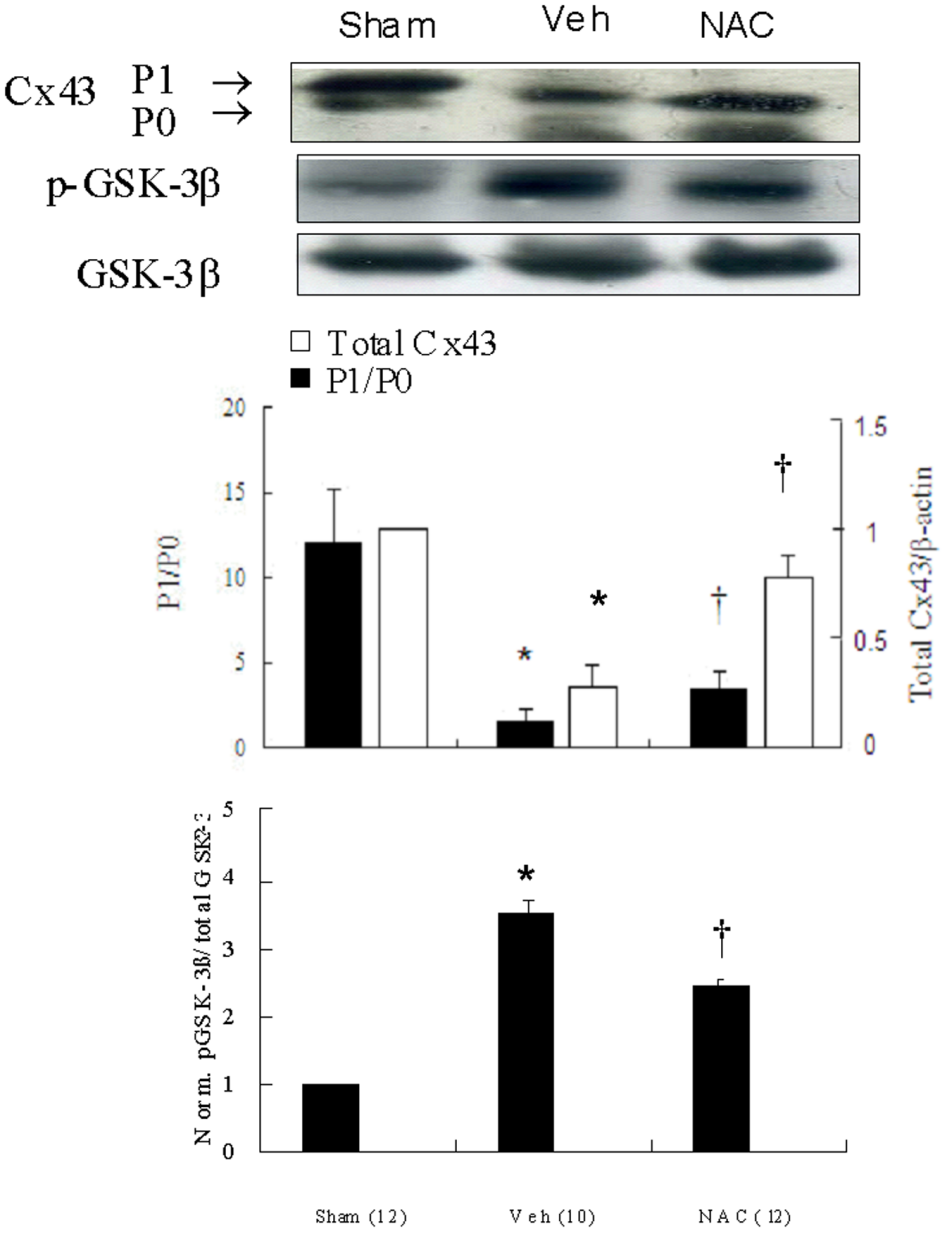
Western blot analysis of Cx43 and GSK-3β. Ventricular remodeling after MI was associated with marked decreased amount of phosphorylated Cx43 (P1). Significantly increased Cx43 amount and P1/P0 ratio had taken place in the NAC-treated group compared with vehicle (Veh). However, a significantly decreased p-GSK-3β is noted in the NAC-treated group compared with vehicle. Relative abundance was obtained by normalizing the density of Cx43 protein against that of β-actin. Densitometric quantification of phosphorylation levels was expressed as the ratio of the density of phosphorylated band over total GSK-3β. Each point is an average of 3 separate experiments. P0, nonphosphorylated Cx43; P1, phosphorylated Cx43. *p<0.05 compared with sham and NAC-treated group; †p<0.05 compared with sham.

Western blot showed that ventricular remodeling is associated with progressively decreased amount of phosphorylated- and total Cx43 at the border zone (Figure 2). In the NAC-treated group, total Cx43 amount was maintained at 68% of that in the sham group, a 45% higher than that in the vehicle-treated group. Furthermore, P1/P0 ratio was significantly increased in the NAC-treated groups compared with vehicle (3.4±0.3 vs. 1.5±0.3, p<0.001). Western blot data were consistent with immunohistochemical data analysis.

### NAC improved the gap junction permeability

**Figure 3:**
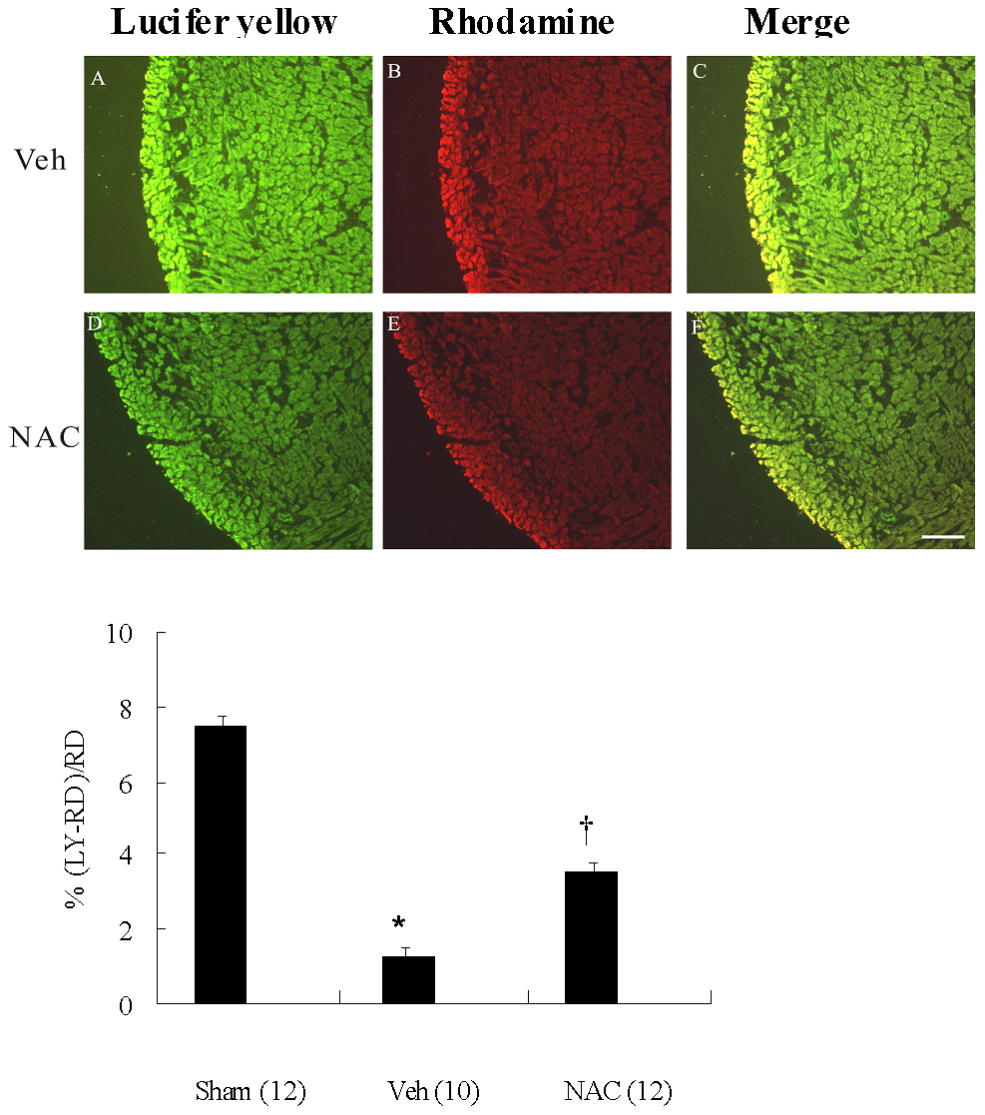
*In vivo* effect of NAC on gap junctional communication. Ventricular remodeling after MI was associated with marked decreased gap junctional communication, which was significantly increased after NAC administration. LY, Lucifer yellow; RD, rhodamine-conjugated dextran; Veh, vehicle. *p<0.05 compared with sham and NAC-treated group; †p<0.05 compared with sham.

As shown in Figure 3, NAC-treated infarcted rats showed a significantly increased gap junction permeability compared with rats treated with vehicle.

### NAC increased Cx43 mRNA

**Figure 4:**
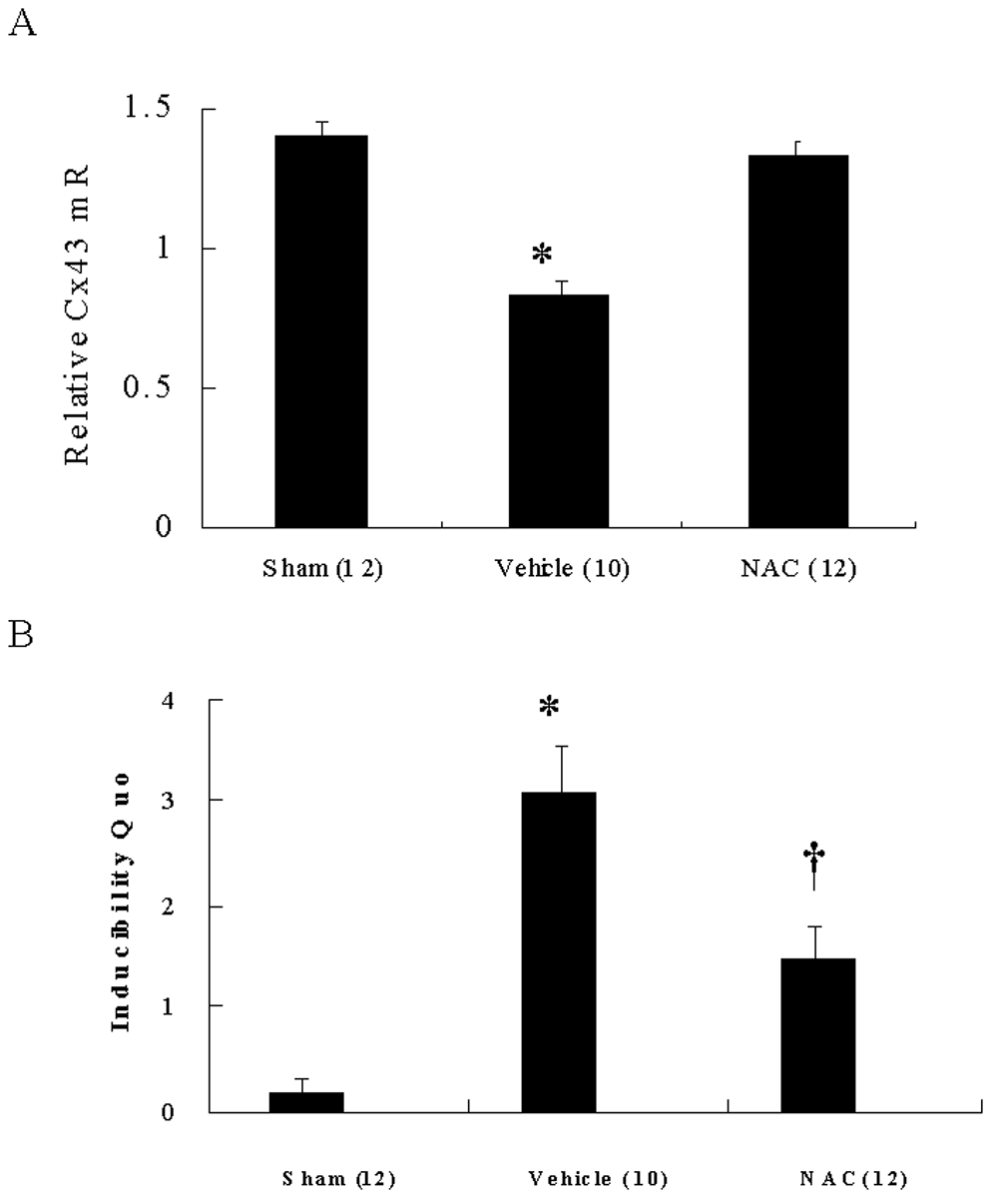
Cx43 mRNA expression and ventricular arrhythmias. **A,** LV Cx43 mRNA levels. Each mRNA was normalized to an mRNA level of cyclophilin. The number of animals in each group is indicated in parentheses. *p<0.05 compared with sham and NAC-treated group. **B,** Inducibility quotient of ventricular arrhythmias by programmed electrical stimulation 4 weeks after MI. The number of animals in each group is indicated in parentheses. *p<0.05 compared with sham and NAC-treated group; †p<0.05 compared with sham.

To determine whether the preserved Cx43 at the border zone of NAC-treated rats was due to an upregulation at the mRNA levels, Cx43 mRNA expression was quantified by real-time RT-PCR. The Cx43 mRNA expression showed a significant downregulation at the border zone in the vehicle compared with sham (Figure 4A). In NAC-treated infarcted rats, the Cx43 mRNA expression was significantly increased than those in the vehicle.

### NAC attenuated pacing-induced arrhythmias

To further elucidate the physiological effect of enhanced Cx43 levels, ventricular pacing was performed. Arrhythmia score in sham-operated rats was very low (0.17±0.11, Figure 4B). In contrast, ventricular tachyarrhythmias consisting of ventricular tachycardia and ventricular fibrillation were inducible by programmed stimulation in infarcted rats. NAC significantly decreased the inducibility of ventricular tachyarrhythmias compared with vehicle.

### cAMP-regulated PKA and Epac augment NAC-induced Cx43 levels

**Figure 5:**
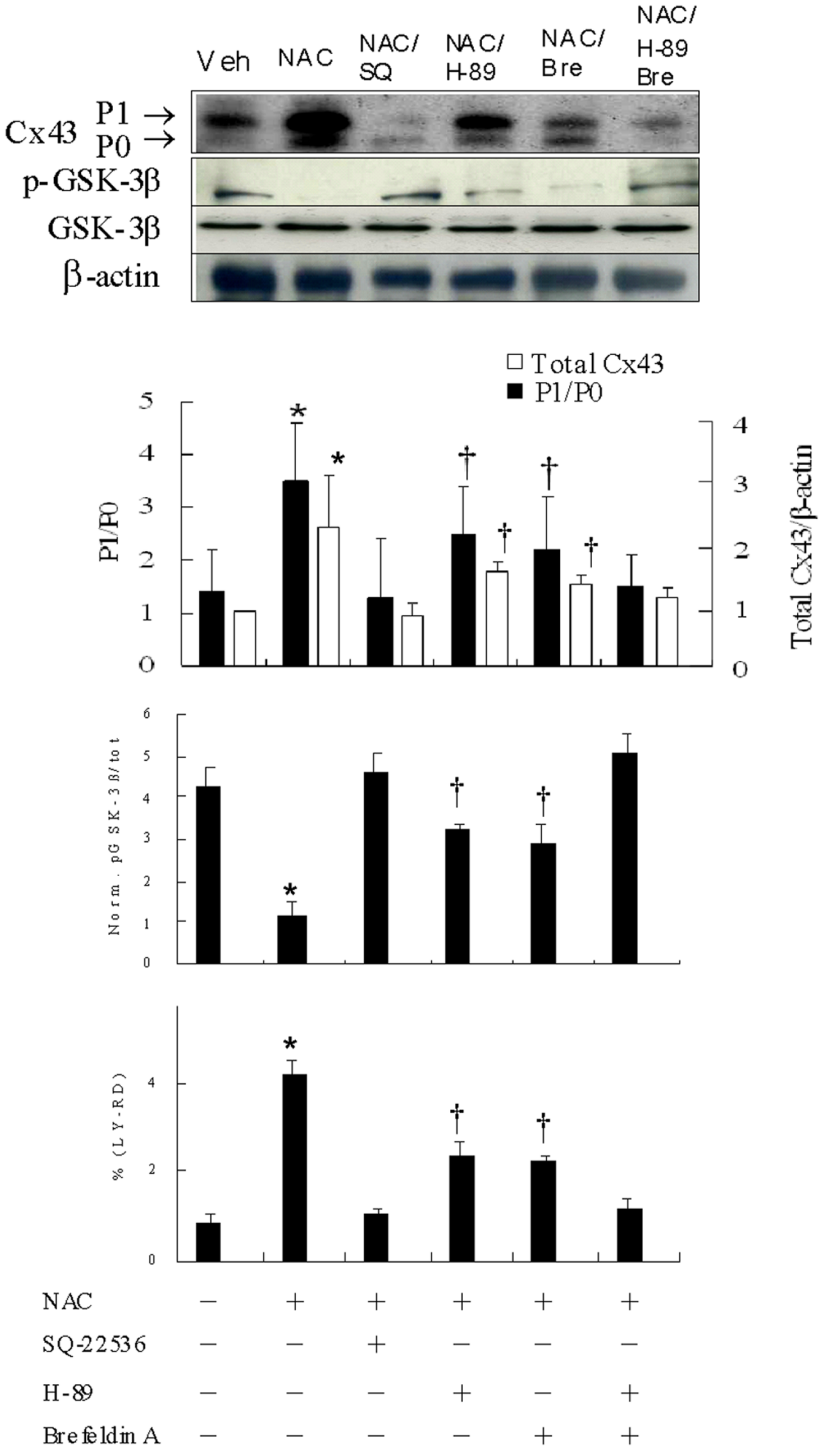
cAMP-regulated PKA and Epac augment NAC-induced Cx43 levels in Experiment 2. In a rat isolated heart model, effect of PKA and Epac was assessed on gap junctional communication and Western blot using antibody against Cx43 and GSK-3β. Significantly decreased Cx43 amount, P1/P0 ratio, and gap junctional communication are noted in the groups treated with SQ-22536, H-89 and brefeldin A compared with NAC alone. Compared with vehicle (Veh), NAC administration showed significantly decreased p-GSK-3β, which can be partially reversed after adding either H-89 or brefeldin A. SQ-22536 significantly reduced augmentation of NAC-induced Cx43 levels to a much greater extent than either H-89 or brefeldin A alone. Relative abundance was obtained by normalizing the density of Cx43 protein against that of β-actin. Each point is an average of 3 separate experiments (n = 10 per group). LY, Lucifer yellow; P0, nonphosphorylated Cx43; P1, phosphorylated Cx43; RD, rhodamine-conjugated dextran. *p<0.05 compared with groups treated with vehicle, NAC+SQ-22536, NAC+H-89, NAC+brefeldin A, and NAC+H-89+brefeldin A; †p<0.05 compared with groups treated with vehicle, NAC+SQ-22536, and NAC+H-89+brefeldin A.

Western blot findings showed that the Cx43 protein levels were augmented by NAC compared with vehicle in a rat isolated post-infarcted heart model (Figure 5). The adenylate cyclase inhibitor SQ-22526 inhibited the NAC-induced Cx43 levels, implying the important role of cAMP in increasing Cx43 levels. These results are in agreement with previous reports [32] and validated the assays we used in this study.

To study whether cAMP-regulated PKA and Epac might cooperate to augment NAC-induced Cx43 levels, we perfused the infarcted hearts with H-89 and brefeldin A (Figure 5). Compared with NAC alone, PKA inhibitor significantly reduced the amount of total Cx43 induced by NAC to 69%. The partial effectiveness of PKA inhibitor in blocking NAC-induced Cx43 accumulation pointed to a role of another pathway, Epac signaling, in addition to PKA. Intriguingly, Epac inhibitor significantly reduced augmentation of NAC-induced Cx43 levels to a similar extent compared with H-89 (31% vs. 39%, p = 0.52). The gap junction permeability changed in parallel to the Cx43 levels. Besides, compared with vehicle, NAC administration showed significantly decreased p-GSK-3β levels, which can be partially reversed after adding either H-89 or brefeldin A.

### Role of GSK-3β in cAMP-dependent Cx43 levels

**Figure 6:**
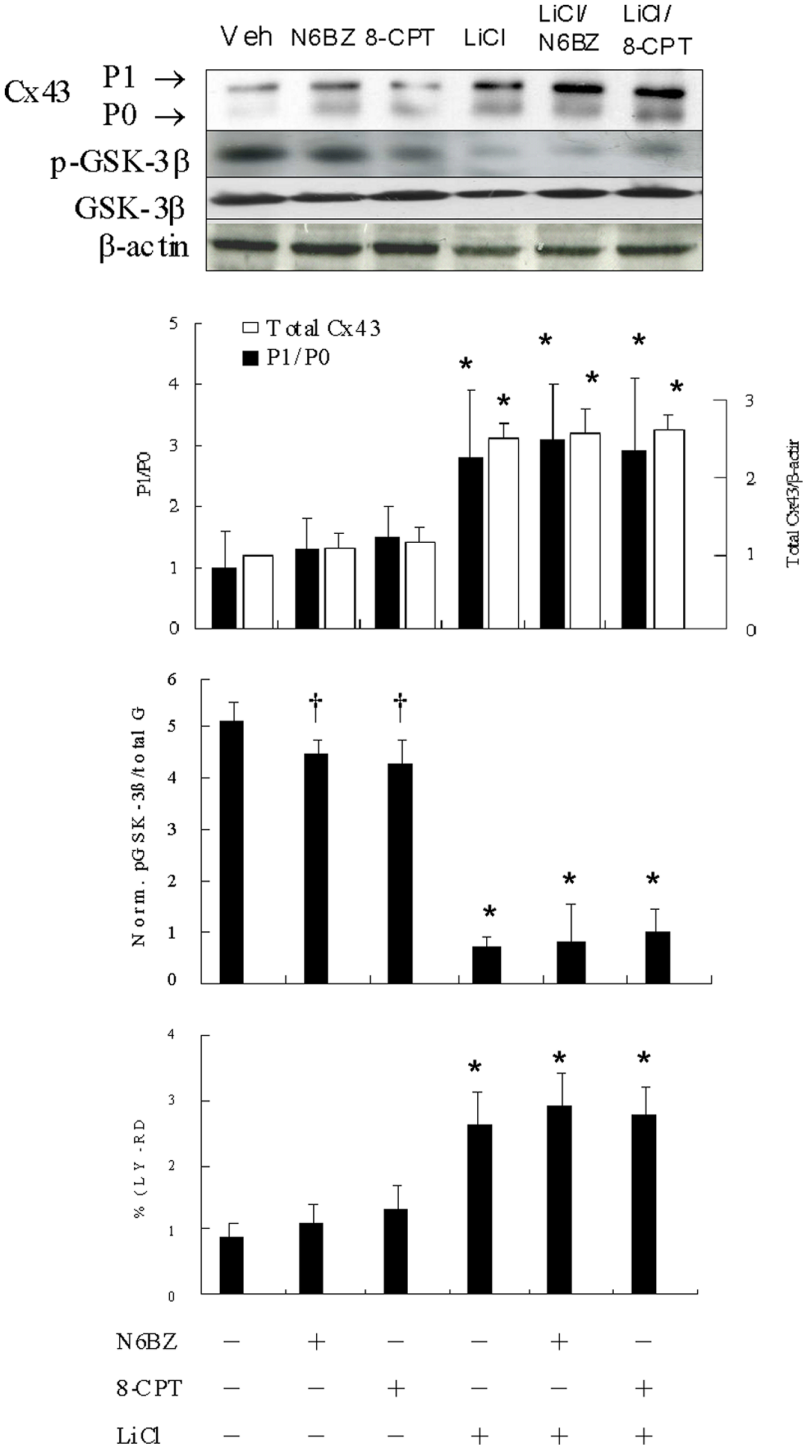
Role of GSK-3β in cAMP-dependent Cx43 levels in Experiment 3. In a rat isolated heart model, effect of GSK-3β was assessed on gap junctional communication and Western blot using antibodies against total Cx43, and phospho- and total-GSK-3β. Significantly increased Cx43 amount, P1/P0 ratio, and gap junctional communication are noted in groups treated with LiCl, LiCl+N6Bz, and LiCl+8-CPT compared with vehicle. Relative abundance was obtained by normalizing the density of Cx43 protein against that of β-actin. Each point is an average of 3 separate experiments (n = 5 per group). LY, Lucifer yellow; P0, nonphosphorylated Cx43; P1, phosphorylated Cx43; RD, rhodamine-conjugated dextran. *p<0.01 compared with groups treated with vehicle, N6Bz, and 8-CPT; †p<0.05 compared with vehicle.

To investigate whether GSK-3β is required for the Epac- and PKA-mediated augmentation of Cx43 levels, we first studied the phosphorylation of GSK-3β activity by N6Bz and 8-CPT. As shown in Figure 6, activation of either Epac or PKA induced markedly decreased phosphorylation of GSK-3β compared with vehicle. Administration with either N6Bz or 8-CPT can not significantly increase the Cx43 levels and dye coupling. Furthermore, addition of either N6Bz or 8-CPT did not have additional increased Cx43 levels compared with rats treated with LiCl alone.

## Discussion

Our present study shows for the first time that chronic treatment for 4 weeks with NAC leads to preserved Cx43 phosphorylation, total amount and function probably through a cAMP-dependent GSK-3β pathway and attenuates ventricular arrhythmias in infarcted rats. The elevation of cAMP by NAC activated two different signaling pathways, namely PKA-dependent and Epac-dependent pathways, in which suppression of GSK-3β and subsequent increased levels of Cx43 are involved. These results were concordant for beneficial effects of NAC, as documented structurally by increase in immunofluorescence-stained Cx43, molecularly by myocardial Cx43 protein and mRNA levels, biochemically by tissue cAMP levels, functionally by dye coupling, and electrophysiologically by improvement of fatal ventricular tachyarrhythmias. Our results were consistent with the notion that cAMP enhanced Cx43 assembly in terms of Cx43 phosphorylation and content [33].

**Figure 7:**
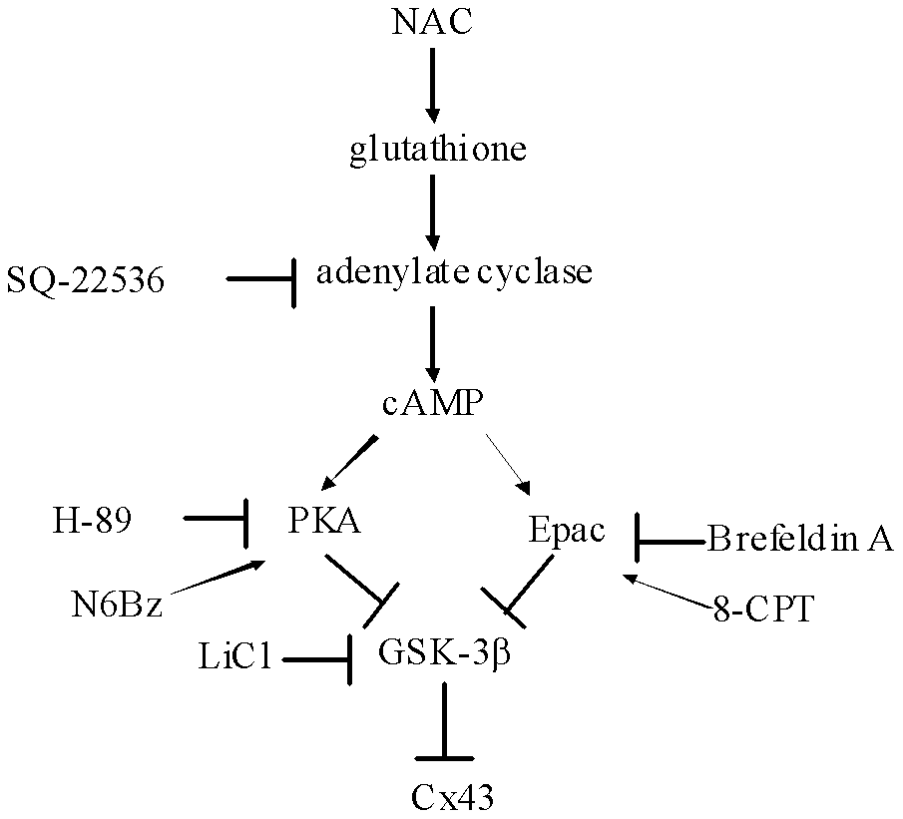
Model for the NAC signaling leads to the increase of Cx43 expression. The adenylate cyclase/cAMP signaling activates both PKA and Epac pathways resulting in phosphorylation of GSK-3β to act as a repressor for Cx43 gene transcription. Inhibition of these signaling pathways by their respective inhibitors is indicated by the *vertical lines* in reference to the direction of the *arrows*.

The present study illustrates an additional role of NAC, showing that NAC is capable of modulating a variety of signal transduction pathways and leads to preserve the phosphorylated form of Cx43. The beneficial effect of NAC on arrhythmias was supported by 3 lines of evidence (Figure 7):

1. NAC administration increased myocardial cAMP levels. It is demonstrated for the first time that administration of NAC is associated with significant increase in the levels of cAMP in infarcted hearts. Our results were consistent with the findings of Smyrniotis et al. [20], showing that NAC administration increased levels of ischemic tissue cAMP in a hepatic ischemia-reperfusion rat model. Recently, we have demonstrated that oxidative stress was increased and GSH levels were significantly reduced after MI [34]. Under conditions of oxidative stress, the thiols in cysteine residues within proteins are among the most susceptible oxidant-sensitive targets and can undergo various reversible and irreversible redox alterations in response to free radicals increase. Decreased content of reduced GSH appeared to play a substantial role in the deficiency of signaling events such as cAMP formation. Previous studies have shown that GSH stimulated adenylate cyclase via stimulatory Gs proteins in patients with Alzheimer's disease [35]. Our results are consistent with previous findings [35], showing that NAC, a GSH precursor, may increase cAMP concentrations by activating adenylate cyclase after MI.
2. cAMP enhanced the Cx43 levels through both PKA and Epac pathways. Our present findings showing that PKA inhibitor is partially effective in reducing Cx43 levels, while the inhibitor of adenylate cyclase significantly further reduces the response, go a step further to indicate that cAMP-potentiating effects of NAC-mediated Cx43 is PKA independent. These findings led us to search for the possible involvement of the recently described cascade mediated by Epac. The concurrent activation of Epac even by increased cAMP significantly enhances downstream signaling. Our study showed that the attenuated degree of Cx43 protein by PKA inhibitor is similar to that by Epac inhibitor. It is likely that the Epac pathway may play a similar role with PKA signaling in regulating Cx43 levels in response to NAC. Our results were consistent with the findings of Somekawa et al [9], showing that Epac predominantly stimulates the accumulation of connexons, whereas PKA regulates gating.
3. GSK-3β functions downstream of PKA and Epac to increase Cx43 levels. Addition of the PKA inhibitor H-89 and Epac inhibitor brefeldin A increased GSK-3β activities and attenuated Cx43 levels compared with rats treated with NAC alone, implying a common pathway of PKA, Epac and GSK-3β in Cx43 regulation. Distinct intracellular cAMP signaling compartments have been recently identified in primary cultures of neonatal cardiac ventriculocytes [36] and cAMP-responsive multiprotein complexes including PKA and Epac seem to confer signaling specificity [37], [38]. cAMP induces GSK-3β phosphorylation via Akt activation in COS cells [39], and HEK293 cells [40]. However, in contrast, other reports show that cAMP-induced phosphorylation of GSK-3β can be mediated via PKA in BHK cells [41] and neurons [42]. Together these results indicate that the relative role of PKA and Epac in GSK-3β phosphorylation may depend on cell types. Our studies provide the first evidence for direct modulation of GSK-3β activity by both PKA and Epac in myocardium. GSK-3β phosphorylation at Ser-9 has been used as an indirect marker of decreased GSK-3β activity. This observation implies that NAC-induced parallel activations of PKA and Epac signaling converge to act together to inhibit GSK-3β activity.

Although activation of either PKA or Epac by N6Bz and 8-CPT induced significantly decreased phosphorylation of GSK-3β, Cx43 levels were not significantly changed, seemingly contrast to our hypothesis. The decrease in GSK-3β phosphorylation by treatment with N6Bz and 8-CPT was significant but modest (12% and 16%) compared with that by NAC (31%, p<0.05). GSK-3 activity has been to be tightly regulated to maintain biological effects. Thus, it is possible that GSK-3 activity is inhibited by PKA and Epac activators alone below the activation threshold of the downstream cascade of Cx43.

### Study limitations

There are some limitations in the present study that have to be acknowledged. First, previous studies showed many contradicting findings regarding the effect of Cx43 phosphorylation on the gating of gap junction channels [13], [43]. Several authors report that phosphorylation of Ser368 contributes to a decrease in dye coupling in cultured fibroblasts [43]. In contrast, phosphorylation of Ser364 has been reported to increase gap junctional communication [13]. TenBroek et al. [13] have shown that cAMP administration increased Cx43 phosphorylation at Ser364. Thus, it is not surprising in our study that NAC attenuated ventricular arrhythmias, in part, by preserving Cx43 phosphorylation through activation of cAMP. Second, Cx43 has nine cysteine residues, three in each of the two extracellular loops and three in the cytoplasmic C-terminal domain. Cysteine residues are susceptible to oxidation, which may be involved in the regulation of the opening of Cx43. Reaction with oxidized GSH (S-glutathionylation) is a protein modification that can occur under oxidative tress. Previous studies have shown that GSH enhanced Cx43 activity when it was applied intracellularly [44]. It has been suggested that NAC increases intracellular GSH either by being converted to cysteine, a precursor of GSH, or by reducing extracellular cystine to cysteine which is more efficiently transported into cells [45]. The increased Cx43 levels in response to NAC may result from direct GSH-dependent increase of the proteins. Finally, the signaling pathways involved were examined pharmacologically. We can not exclude the nonspecific actions of drugs. For example, GSK-3β has been shown to be redox sensitive [46]. Whether NAC directly inhibited the levels of GSK-3β by acting GSH supplement independent of PKA and Epac remained unknown. However, in spite of lack of antioxidation, both the PKA activator and the Epac activator inhibited the levels of GSK-3β. It suggested that factors other than redox may contribute to the pathogenesis of attenuated levels of NAC-related GSK-3β. To further confirm the role of NAC-induced GSK-3β levels, specific genetic approaches are warranted such as transfection GSK-3β mutant to interfere the enhanced effects of cAMP on Cx43 levels.

### Conclusions

PKA and Epac are the essential signaling pathways responsible for NAC-mediated Cx43 levels. The significance of the finding that NAC modulation of Cx43 levels may have relevance for strategies to regulate Cxs may provide a novel means to diminish ventricular arrhythmias associated with NAC use. These effects are functionally and structurally important because they are linked to attenuated incidence of fatal arrhythmias. Our study provides a new insight into the mechanisms responsible for the pharmacological activity of NAC and suggests a new strategy for the prevention of postinfarction ventricular arrhythmias.
